# Epidemiological investigation and prevention and control strategies of rubella in Anhui province, China, from 2012 to 2021

**DOI:** 10.3389/fpubh.2022.991799

**Published:** 2022-10-06

**Authors:** Ning Zhang, Xiaodong Cheng, Shujie Zhou, Binbing Wang, Xianwei Luo, Yu Chai, Jihai Tang, Bin Su, Zhirong Liu

**Affiliations:** Anhui Provincial Center for Disease Control and Prevention, Hefei, China

**Keywords:** rubella, epidemiology, elimination, prevention and control, strategies

## Abstract

**Background:**

Rubella is a highly contagious viral infection with mild manifestations that occurs most often in children and young adults. Infection during pregnancy, especially during the first trimester, can result in an infant born with congenital rubella syndrome (CRS). The purpose of this paper is to analyze the characteristics of rubella epidemics in Anhui province from 2012 to 2021 and explore the prevention and control strategies of rubella.

**Methods:**

A descriptive epidemiological approach was used to examine the epidemiological characteristics of rubella in Anhui Province between 2012 and 2021.

**Results:**

From 2012 to 2021, a total of 4,987 cases of rubella were reported in Anhui province, with an average annual incidence of 8.11 per million, demonstrating an overall downward trend (χ^2^ trend =3141.06, *P* < 0.01). The average yearly incidence of rubella in southern Anhui, central Anhui, and northern Anhui were 9.99 per million, 11.47 per million, and 4.50 per million, respectively, with statistically significant differences (χ^2^ =792.50, *P* < 0.01). The male to female incidence ratio was 1.67:1, and the male incidence rate was higher than the female incidence rate. Most cases occurred among students, accounting for 56.59% of all cases, and the 10–34 age group accounted for ~73.71% of all cases. Regarding immunization history, 3.57% of cases had two doses or more, 6.62% had one dose, 16.40% had none, and the remainder were uncertain.

**Conclusion:**

The incidence of rubella in Anhui province from 2012 to 2021 continued to decline, with regional variations observed. The 10–34-year-old population without a history of rubella vaccination is at high risk for the disease. It is suggested to carry out rubella vaccination and congenital rubella syndrome monitoring according to the actual situation.

## Introduction

Rubella is a highly contagious viral infection with mild manifestations that occurs most often in children and young adults (1). It is caused by the rubella virus (RV), transmitted by airborne droplets when infected individuals sneeze or cough. Infection during pregnancy, especially during the first trimester, can result in miscarriage, fetal death, stillbirth, or congenital malformations in infants, known as congenital rubella syndrome (CRS). Over 100,000 children are born each year with CRS worldwide (2).

In 2012, the World Health Assembly signed the Global Vaccine Action Plan (GVAP), which proposes to eliminate measles and rubella in at least five WHO regions by 2020 (3). As a result of rubella-containing vaccine (RCV) vaccination programs, disease incidence has declined substantially in many countries. 70% of the world's infants were vaccinated against rubella in 2020. The reported rubella cases decreased by 48% from 94,277 in 2012 to 10,194 in 2020. Rubella elimination has been verified in 93 countries, including the entire Region of the Americas (AMR) (4). The preferred strategy for introducing RCV into national immunization programs has been used to eliminate rubella and CRS in AMR, targeting the majority of people who may not have been naturally exposed to rubella (usually children and adolescents under 14 years of age) (5).

RCV became available in China in 1993 and was introduced nationwide under the Expanded Immunization Program (EPI) in 2008. Since then, rubella incidence has dropped significantly (6). However, rubella cases continued to rise in China from 2018 to 2019, and people aged 10–29 without a history of rubella immunization were at risk (7). In order to eliminate rubella, research into the nature of rubella epidemics and the precise prediction of rubella epidemic trends are crucial.

Located in central and eastern China, Anhui province consists of 16 cities and has a total area of 140,100^2^ km, which accounts for ~1.45% of China's total surface area. By the end of 2021, the province had 61.13 million permanent residents. From north to south, Anhui province is divided into three regions: The northern region includes Bengbu, Huainan, Huaibei, Suzhou, Fuyang, and Bozhou; the middle region has Hefei, Lu'An, Chuzhou, and Anqing; and the southern region include Wuhu, Ma'Anshan, Tongling, Xuancheng, Chizhou, Huangshan. As far as we are aware, this is the first study to analyze a rubella epidemic over ten consecutive years in the Anhui province. We aimed to analyze the epidemiological characteristics of rubella in Anhui Province from 2012 to 2021, identify the weaknesses in rubella prevention and control, and develop a scientific basis for the prevention, control, and elimination of rubella.

## Materials and methods

### Data source

#### Rubella surveillance

In 2004, the National Notifiable Diseases Reporting System (NNDRS) was established, a web-based real-time reporting system for mandatorily reported infectious diseases including rubella. In 2014, rubella was integrated into the measles surveillance system in China. NNDRs passively collects and transmit individual-level case data information countrywide, Surveillance data flowed to the national CDCs step by step from hospitals and county-level CDCs. In measles surveillance system, hospitals report measles or rubella suspected cases, county CDCs conduct epidemiological investigation and input the results, city and provincial CDCs conduct specimen testing and input the results, while the case information flow to the national CDCs step by step. Integrated Management System contains data such as demographic information reported step by step. The above three systems and National Public Health Emergencies Reporting Information System are subsystems of China disease prevention and control information system.

Cases of rubella were obtained from the measles and rubella surveillance information system from 2012 to 2021, whose address was Anhui Province. A uniform questionnaire to gather disease and epidemiological data was used to investigate every suspected rubella case. Information collected includes name, gender, birthday, occupation, onset date, case classification, vaccination doses, etc. Demographic data, which were required for calculating the incidence of rubella, were obtained from the Integrated Management System for Disease Prevention and Control. Rubella emergency information was derived from the National Public Health Emergencies Reporting Information System. At present, China does not have a CRS surveillance system.

#### Routine immunization coverage of RVC

Government-operated vaccination clinic deliver all vaccines to locally born children and children of new arrivals who have registered for clinic services. Each dose of vaccine is recorded by Clinic immunization providers in clinic medical records, Child's Vaccination Certificate held by parents, and a computerized immunization information system. Clinics calculate and report EPI vaccination coverage to county CDCs by dividing the number of children vaccinated in the clinic with a specifically required dose or dose sequence of vaccine by the number of children registered in the clinic. The number of children vaccinated with a specifically required dose or dose sequence of vaccine is taken as the molecule, while the number of all children registered at the clinic is used as the denominator. Data are aggregated from vaccination clinics through the routine immunization information system step by step, ultimately to China CDC.

In our study, routine immunization coverage for the first dose of RCV (RCV1) and the second dose of RCV (RCV2) were calculated from 2009 to 2021, obtained by the routine immunization information system.

### Case definitions

A case-based, laboratory-supported measles-rubella surveillance program is conducted in all cities in Anhui province. The suspected case can be defined as a person with a fever, rash, cough, coryza, conjunctivitis, lymphadenopathy, arthritis, or arthralgia, and any measles or rubella case questioned by the health worker responsible for the infectious disease.

**Figure 1:**
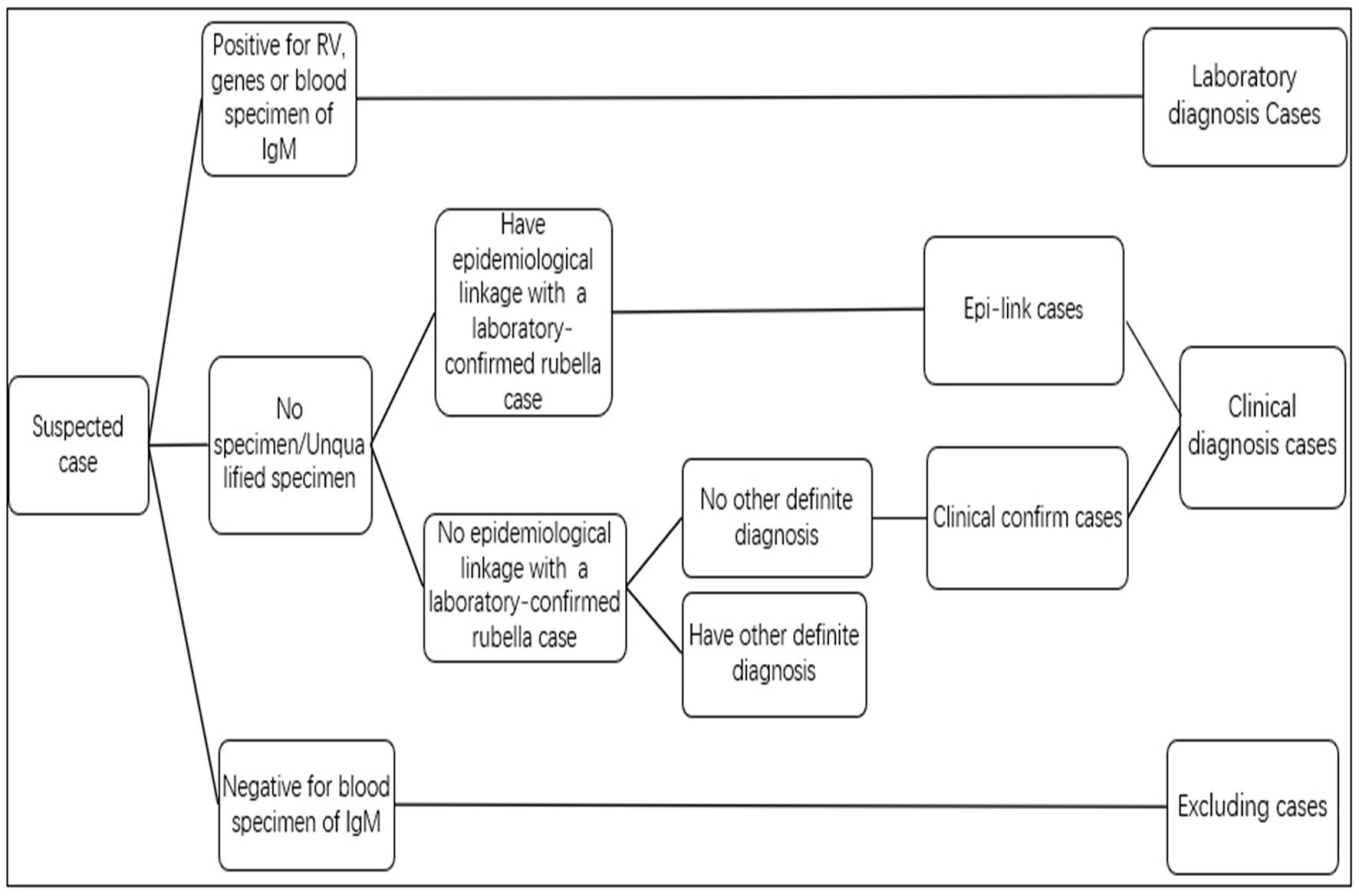
Standard of the rubella suspected case classification.

Rubella cases were diagnosed and classified by the national monitoring scheme of measles promulgated by the ministry of health (Figure 1). This study includes laboratory and clinical diagnoses of rubella cases.

### Ethical review

Rubella surveillance data collection and analysis are considered routine public health activities by Anhui CDC's Ethical Review Committee and do not require specific approval from the ERC.

### Statistical analysis

This study utilized descriptive epidemiology to analyze the epidemiological characteristics of the rubella epidemic in Anhui Province between 2012 and 2021, including the main timeframe, regions, and population. Data collection and statistical analysis were conducted using Excel 2010 and SPSS 23.0 statistical software. Qualitative data were described using frequency descriptions. Chi-square test and Chi-square trend test were used for comparison of incidences of rubella.

## Results

### Annual incidence

**Figure 2:**
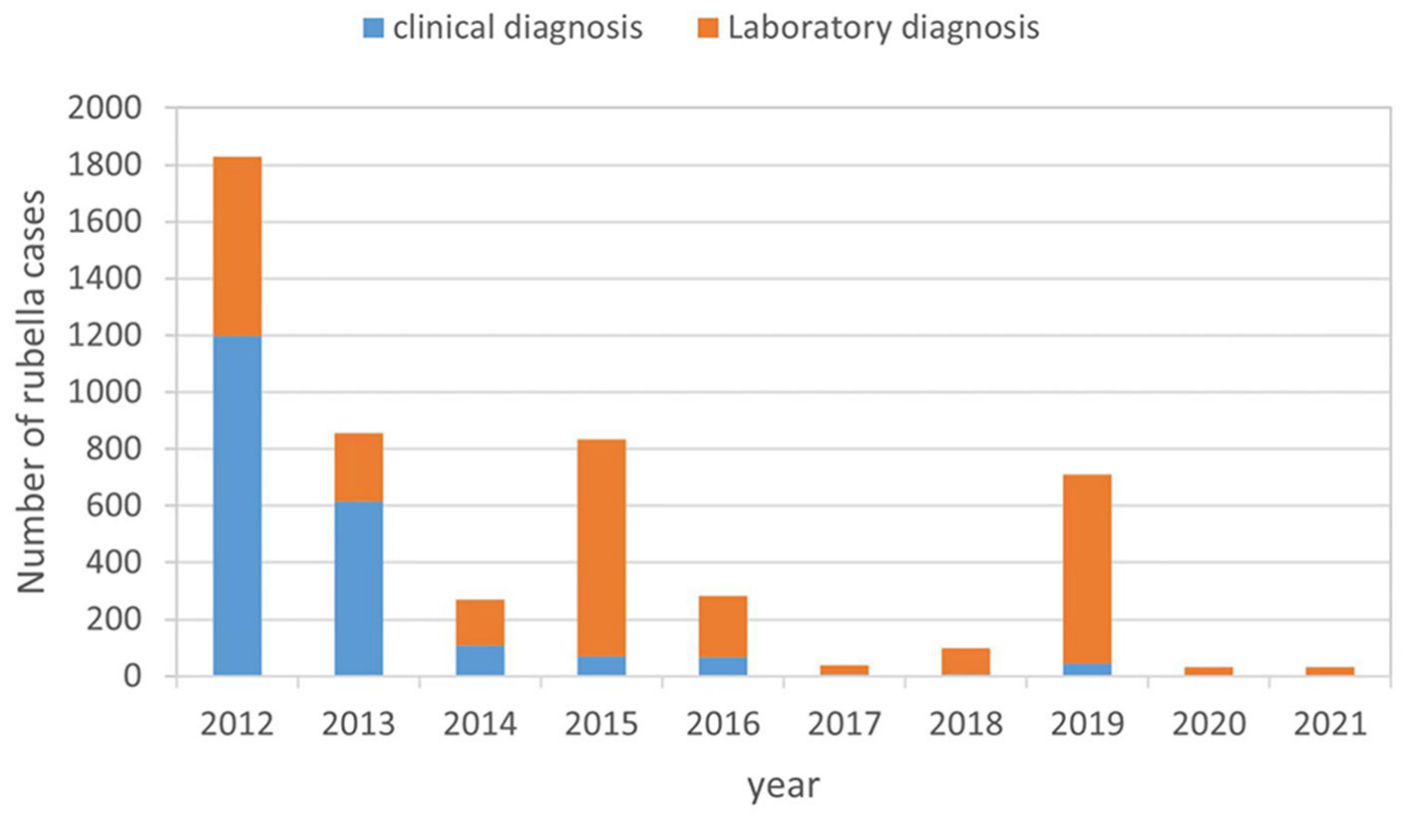
Rubella surveillance performance in Anhui province from 2012 to 2021.

From 2012 to 2021, 4,987 cases of rubella have been reported in Anhui Province, with an average annual incidence of 8.11 per million. The incidence of rubella declined from 30.65 to 4.49 per million between 2012 and 2014, recovered to 13.73 per million in 2015, and then decreased significantly to 0.65 per million in 2017. As the year progressed, the rubella incidence slowly recovered. A small peak with an incidence of 11.23 per million occurred in 2019. Since 2020, the incidence has declined significantly and remained low at 0.54 per million from 2020 to 2021, 98.37% lower than in 2012. The proportion of laboratory diagnosis cases was 32.44% (871/2685) during 2012 to 2013, increased to 59.78% (162/271) in 2014 when rubella was integrated into measles surveillance, continued to increase to 90.00%(1828/2031) during 2015 to 2021 (Figure 2). From 2014 to 2021, 25178 measles-rubella suspect cases were reported including 17908 non-measles, non-rubella cases, and the annual reporting of non-measles, non-rubella cases per 100 000 population ranged from 2.52 to 7.12.

### Month distribution

Rubella cases were reported throughout the whole year in Anhui province. The number of cases showed evident seasonal distribution from 2012 to 2018. The peak was observed from March to June, with rubella cases accounting for 75.62% (3722/4922) of the total cases. Cases are rare and sporadic throughout the year, with no obvious seasonality from 2020 to 2021.

### Regional distribution

Among 4,987 rubella cases, Central Anhui reported the most significant number, 50.43% (2515/4987) of the total cases. In addition, Southern Anhui and Northern Anhui accounted for 25.23% (1258/4987) and 24.34% (1214/4987), respectively. Based on the data collected between 2012 and 2021, the average annual incidence of rubella in Southern Anhui, Central Anhui, and Northern Anhui was 9.99, 11.47, and 4.50 per million, respectively, with statistically significant differences (c^2^ = 792.50, *P* < 0.01). Compared to the province as a whole, southern Anhui and Central Anhui had higher incidence rates. The Chi-square test was used to analyze the incidences of the three regions year by year, indicating that the incidences of the other 9 years were statistically significant except for 2020 (Table 1, Supplementary Table S1).

**Table 1:** Rubella Incidence by region in Anhui Province from 2012 to 2021 (per million).

**Year**		**Region**		**Anhui**	**χ^2^**	***P*-Value**
	**Northern Anhui**	**Central Anhui**	**Southern Anhui**			
2012	17.04	42.93	36.81	30.65	278.69	<0.001
2013	5.54	24.10	14.77	14.30	287.29	<0.001
2014	2.25	5.97	6.55	4.49	51.16	<0.001
2015	11.80	11.26	22.44	13.73	84.35	<0.001
2016	2.41	3.08	11.60	4.57	177.99	<0.001
2017	0.14	1.03	1.08	0.65	19.50	<0.001
2018	0.96	2.79	1.00	1.60	29.19	<0.001
2019	5.52	20.66	7.93	11.23	267.13	<0.001
2020	0.46	0.64	0.37	0.50	1.35	0.51
2021	0.22	0.91	0.57	0.54	10.61	0.005
Total	4.50	11.47	9.99	8.11	792.50	<0.001

### Age distribution

**Figure 3:**
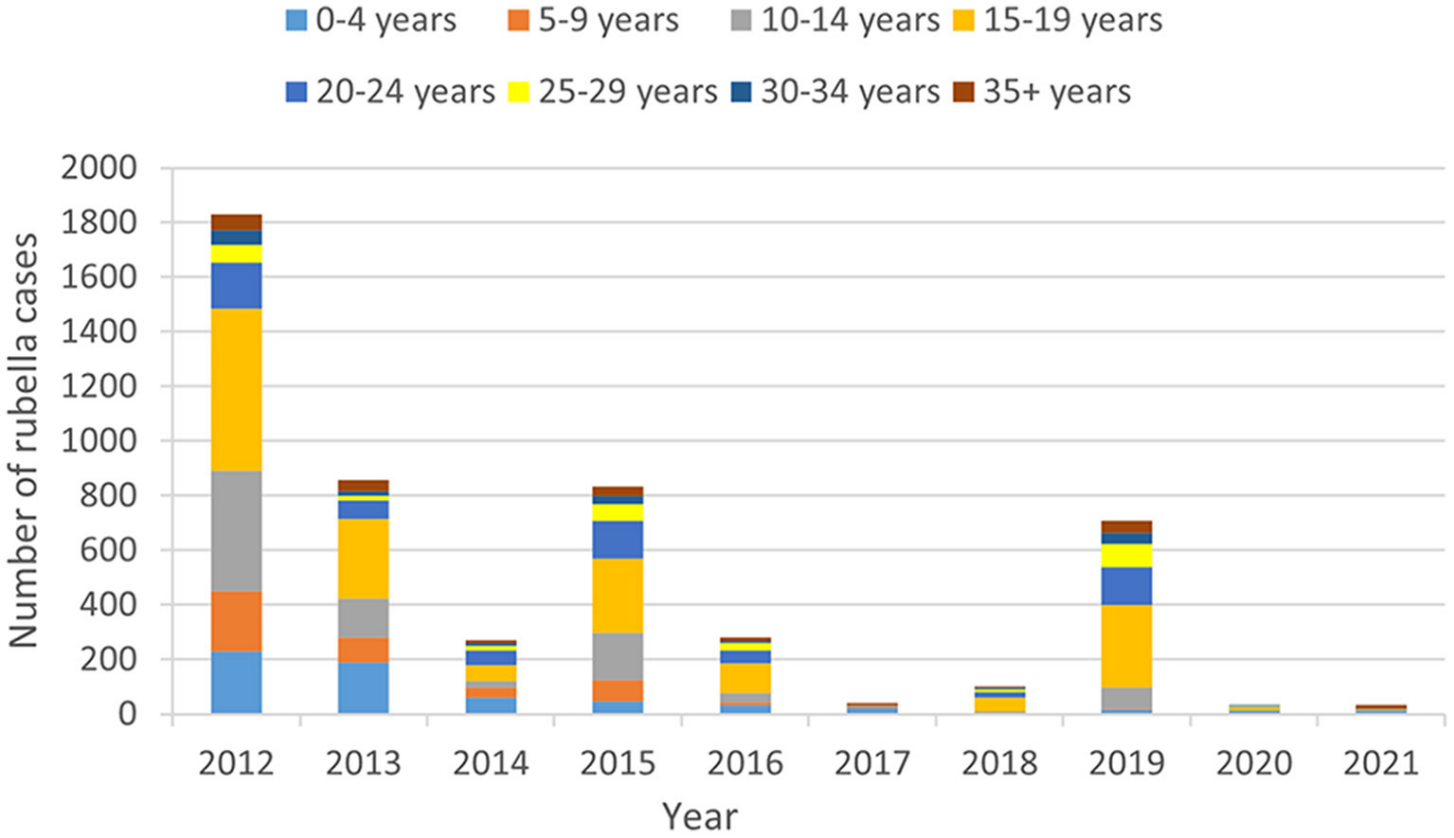
Rubella cases age distribution in Anhui province from 2012 to 2021.

The youngest case was less than 1 year old, while the oldest case was 79 years old. The median age of rubella cases was 16 years. Cases involving individuals aged 10–34 were the majority, accounting for 73.71% (3676/4987) of all cases. The age group 15–19 years old had the highest number of cases, accounting for 33.91% (1691/4987) of the total number of cases. It was followed by the 10–14 age groups, which accounted for 17.91% (893/4987) of the total cases. Figure 3 shows the age distribution varied substantially by year.

### Sex distribution

There were 3,118 male cases and 1,869 female cases, with a male-to-female ratio of 1.67:1. The average annual incidence among males was 10.04 per million, significantly higher than that of females, 6.14 per million (c^2^ = 289.1, *P* < 0.01). Incidences of rubella were higher in men than women in all years except 2017. A year-by-year comparison of the incidence showed that the incidence of males and females were statistically significant in other 6 years except 2017, 2018, 2020, and 2021 (Table 2, Supplementary Table S2).

**Table 2:** The incidence of rubella between male and female from 2012 to 2021 (per million).

**Year**	**Incidence**		**χ^2^ value**	***P*-value**
	**Male**	**Female**		
2012	37.08	24.10	82.02	<0.001
2013	17.92	10.61	55.87	<0.001
2014	5.69	3.26	19.85	<0.001
2015	17.08	10.42	49.06	<0.001
2016	6.12	3.01	32.3	<0.001
2017	0.57	0.72	0.565	0.452
2018	2.06	1.13	8.41	0.004
2019	14.37	8.03	56.51	<0.001
2020	0.62	0.38	1.84	0.175
2021	0.68	0.40	2.12	0.145
Total	10.04	6.14	289.1	<0.001

### Occupation distributing

Among the study cases, students accounted for 56.59% (2822/4987); the proportion of scattered children, workers, famers, and household or unemployed people, was 12.89% (643/4987), 7.20% (359/4987), 7.16% (357/4987) and 4.63% (231/4987) respectively. These contributed 88.47% of the total cases.

### Rubella vaccination status of rubella cases

This study evaluated rubella vaccination status from 2015 to 2021 since this information was only reported after the national monitoring scheme of measles was implemented in 2014. Among the 1,933 cases that had RCV immunization history, 16.40% (317/1933) had no dose, 6.62% (128/1933) had one dose, 3.57% (65/1933) had two doses or more, and 73.62% (1423/1933) had unknown immunization status. There were 46 cases of children under the age of 8 months who were not eligible for vaccination. Among the cases between the age of 8 months and 4 years, 14.85% (15/101) had no dose, 70.30% (71/101) had one dose at least, and 9.90% (10/101) had an unknown vaccination status. In cases of 5–9-year-olds, 10–14-year-olds, and 15–19-year-olds, 24.77% (27/109), 10.86% (33/304), and 5.66% (39/689) had histories of receiving at least one dose of RCV.

### Routine immunization coverage rate of RCV

**Figure 4:**
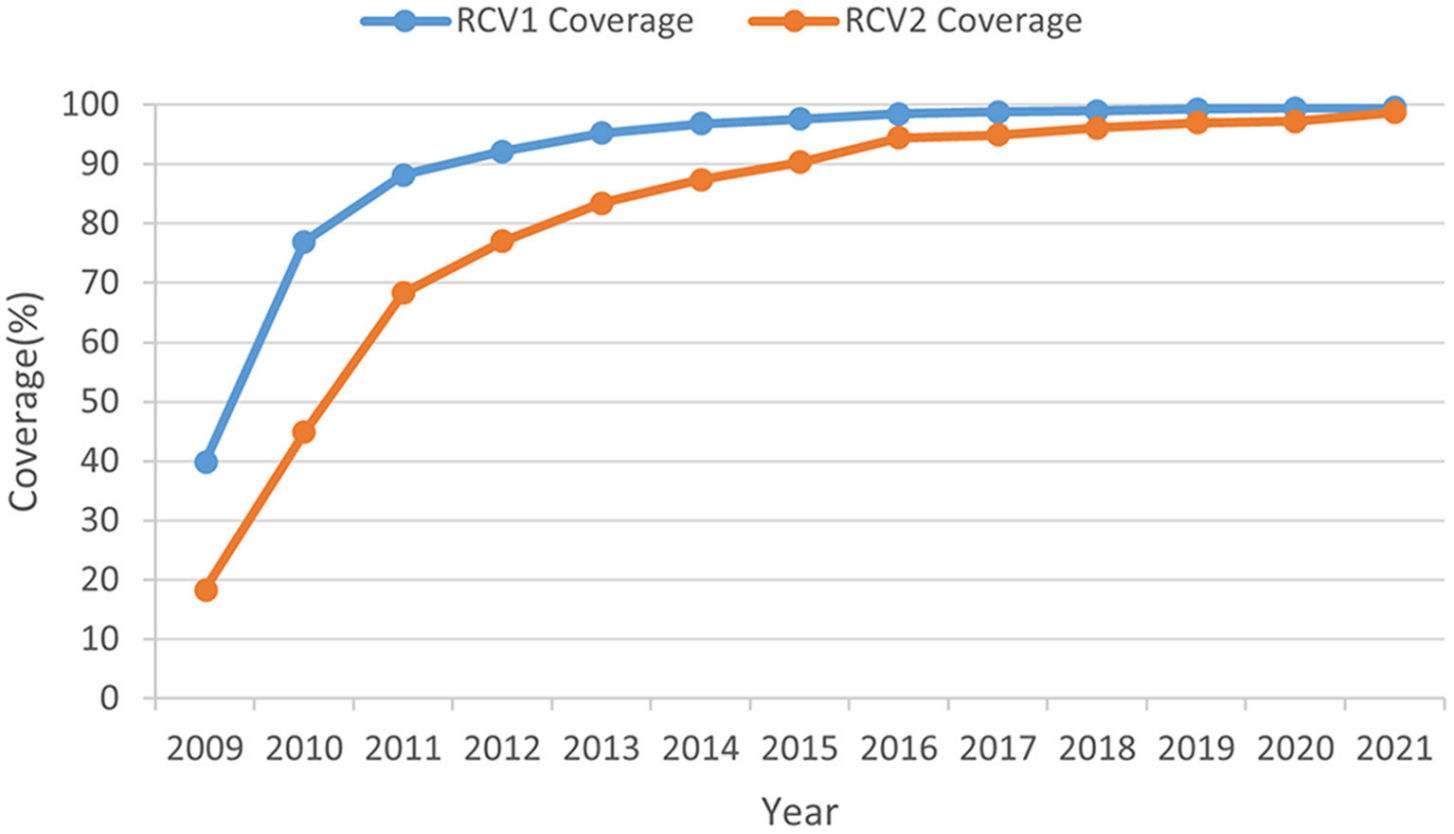
Reported RCV coverage levels in routine immunization from 2012 to 2021.

The reported routine RCV1 coverage was lowest in 2009 (39.75%). Since then, it has risen year by year and maintained above 95% during 2013–2021. The reported routine RCV2 coverage showed the same pattern-lowest in 2009 (18.29%), increasing to 90.29% in 2015, maintaining above 95% from 2018 to 2021 (Figure 4). The reported routine coverage of RCV1 and RCV2 in each city has exceeded 95% since 2015 and 2018 respectively (Figure 4, Supplementary Tables S3, S4).

### Rubella public health emergencies

Between 2012 and 2021, 11 rubella public health emergencies involving 499 cases were reported in Anhui province. These emergency reports were made by Central Anhui (six involving 261 cases), Southern Anhui (four involving 221 cases) and Northern Anhui (one involving 11 cases) respectively. Of the 11 incidents, nine occurred in schools, including seven in middle schools, one in primary school and one in adult colleges, the other two in factories.

## Discussion

Before the use of the RCV vaccine, large rubella epidemics occurred every 3–8 years (2). China included RCV into China's nationwide EPI system in 2008, with a schedule of 1 dose of MR at 8 months and 1 dose of MMR at 18 months. It was an essential step toward controlling and eliminating rubella and preventing CRS, which meant that the vaccines were accessible to children free of charge. There was a significant effect on controlling the transmission of the rubella virus and protecting children of the appropriate age. Rubella incidence in China has declined since the introduction of the rubella vaccine, with the lowest level in 2017, but rebounded and continued to rise in 2018–2019 (6, 7). The epidemic trend of rubella in Anhui province is consistent with that of the whole country, whose incidence of rubella showed a declining trend from 2012 to 2021. There was a small peak in 2015 and 2019 due to the high number of cases involved in public health emergencies with rubella. The growing immunized blank population and the fact that rubella incidence cycles every 6–8 years (8) suggest that Anhui Province might be in the early stages of a new rubella epidemic cycle in 2019. Since 2020, the incidence of rubella has decreased significantly. There was a significant drop in rubella incidence in Anhui province in 2020–2021 to reach the lowest level. Since COVID-19 began spreading in 2020, the public has frequently worn masks, washed hands, maintained social distance, and practiced appropriate hygiene. As a result, the viral spread of many respiratory infections, including rubella, has been controlled. Rubella is difficult to diagnose accurately without laboratory testing, therefor, laboratory diagnosis rate is crucial for rubella elimination. The proportion of laboratory diagnosis of rubella is increasing from 32.44 to 90.00%, indicates that the elimination of rubella in Anhui Province is credible. Since 2014, the annual reporting of non-measles, non-rubella cases per 100 000 population ranged from 2.52 to 7.12, exceeding the sensitivity targets established by the WHO Western Pacific Region. The high sensitivity of measles and rubella surveillance system is an important prerequisite for rubella elimination. As in previous years (9, 10), rubella incidence remains seasonal, peaking between March and June in other provinces and Anhui provinces.

Rubella cases were reported in all cities of Anhui province. Most rubella cases were reported in central Anhui province, whose annual incidence was the highest. Central Anhui should be treated as the key area of rubella prevention and control.

During the first few years after RCV was included into EPI system, the introduction of rubella was accompanied by an understanding that supply may not keep up with demand. Ministry of Health recommended that measle vaccine or measles-mumps vaccine can be used to replace MMR or MR to cope with the insufficient supply of MMR or MR (6). Different needs for vaccine substitution between cities result in different RCV coverage. Based on the routine immunization coverage, although RCV coverage were increasing continuously in all cities, there were still some differences in both RCV1 and RCV2 coverage among cities in each year. Most of the RCV coverage in cities of central Anhui were below the middle level. The difference of RCV coverage might affect the immune level of the population to some certain extent. A serological survey of healthy people in central Anhui showed the positive rate of rubella antibody was 80.8% (11), which was lower than that of women of childbearing age in northern Anhui (12). It is suggested that the population susceptibility in central Anhui is higher. This may be one of the reasons for the high number of outbreaks in Central Anhui. Therefore, improving RCV coverage in the whole population was still the key point of rubella elimination.

In terms of gender distribution, the male to female ratio of rubella cases was 1.67:1, higher than the previous ratio (13), similar to that in Henan Province (10), as opposed to that in Africa (14). The incidence in males is higher than in females, which is consistent with the national study (7). Subsequent studies on the differences in rubella exposure factors between sexes can be conducted.

The study revealed that rubella antibody-positive rates in children were low before RCV was included in EPI, especially in the 10–14 age group (15). Rubella incidence was mainly concentrated in Anhui Province aged 5–14 years old (13). Upon the inclusion of RCV into the standard vaccination of EPI, the age composition of rubella cases changed, with an increase in cases among older individuals. A study in Beijing showed that the onset of rubella mainly occurred at the peak of childbearing age (16). Rubella cases in Anhui province from 2012 to 2021 were concentrated primarily on adolescents and adults aged 10–34, consistent with the age composition of rubella incidence in China (7). Corresponding to age distribution, the occupation distribution of rubella cases among students was the largest.

In Anhui province, RCV has been included in EPI since 2008.The population born before 2008 was not able to obtain antibodies through RCV. In addition, the standardized inoculation of RCV and the gradual establishment of an immune barrier significantly reduce the likelihood of this population acquiring antibodies through natural infection. If such an immunity gap persists for a prolonged period, it is likely to lead to an accumulation of susceptible people, including those who are currently in a baby boom and those who are about to enter one. In other provinces, it was found that adults' immunity levels were much lower than those of children (17–19). Lower population immunity may increase the risk of infection during pregnancy, thereby paradoxically increasing the risk of CRS (20), suggesting that the risk of CRS may increase in Anhui province in the future. 2020–2021 is the year of the low incidence of rubella, and the proportion of people aged 10–34 decreased significantly. This may be due to the fact that COVID-19 prevention and control work may influence these individuals to implement prevention and control measures, such as wearing masks and washing hands frequently, which, in turn, prevents rubella virus transmission. Filling the immunization gap is the best strategy for preventing and controlling rubella in this population. In order to achieve a higher level of immunity, it is recommended that all cities monitor rubella serological antibody levels and vaccinate adolescents and young adults with RCV according to local incidence, vaccine supply, and quality assessment results of vaccination work. The monitoring of rubella antibody levels in women of childbearing age in Beijing and Chongqing city suggests that 14.79–21.47% of childbearing age women are susceptible to rubella virus (21). Most women of childbearing age and about to enter childbearing age in Anhui province have not received RCV, so rubella antibody detection can be considered during the pre-marital examination, and one dose of RCV should be inoculated to women with negative rubella antibody. At the same time, CRS surveillance is also of great significance to quickly identify rubella outbreaks and take timely measures to avoid the spread of the epidemic caused by CRS cases.

According to the national measles surveillance program, the immunization history investigation of measles and rubella cases, especially those under 15 years old, is one of the core contents of epidemiological research. From 2015 to 2021, only 9.98% of rubella cases had a history of RCV vaccination, and the remainder fell into the blank immunization category. Only 9.72% of rubella cases aged 8 months to 14 years had immunization history, 85.34% had 0 doses or unknown immunization history. Consequently, there are still some weak links in routine immunization against RCV among children, and the accumulation of immune blanks over time is the leading cause of rubella cases. Ensuring adequate protection against vaccine-preventable diseases by checking the vaccination status of school children is a national task mentioned explicitly in the legal framework of China's immunization program. Collaboration with the Ministry of Education to strengthen the school vaccination record check and fulfill its statutory requirement may be one means of ensuring the protection of future young adults from rubella. Anhui province needs to strengthen rubella surveillance and school vaccination record check based on routine immunization. Once a rubella epidemic is found, it must be reported in a timely and standardized manner, carry out a convenient and detailed epidemiological investigation under the requirements of the monitoring program, and implement prevention and control measures to prevent the spread of the epidemic.

Nine out of 11 public health emergencies with rubella occurred in schools. Because rubella symptoms are mild while the recessive infection rate is high, it is challenging to recognize recessive infected people and early patients. Still, they are an essential source of infection that cannot be ignored. The school environment is relatively closed, and the population is concentrated. Due to untimely screening, it is easy to trigger outbreaks or epidemics when an infection source enters but no effective measures are taken. Schools should be the fundamental units for rubella prevention and control. CDC should train the school doctors and health care physicians in all schools within their jurisdiction, improve awareness of epidemic reporting, and implement health monitoring, especially during rubella season with high incidence rates, to achieve early detection, early reporting, and early treatment. Currently, most rubella emergencies occur in middle schools, colleges, and universities. Local governments can consider how to carry out RCV vaccination according to the actual situation of colleges and universities, collective units, and other personnel in a planned way to fill the blank of population immunization further.

A significant strength of this study is that it analyzes rubella epidemiological characteristics for ten consecutive years. Furthermore, high-quality data from China's rubella surveillance system was used, including accurate case definition, standardized epidemiological investigation, and uniform surveillance protocols.

However, several limitations in this study should be noted. First, in the survey of RCV immunization history, uncertain accounted for 73.62% (1423/1933), which may affect our results. Rubella, with 30–50% of subclinical cases, is milder than measles. Even though their surveillance systems are integrated, rubella is less likely to be detected than measles.

In summary, rubella incidence varied by region in Anhui province. Immune blanks between the ages of 10–34 are at high risk for rubella. Rubella vaccination should be carried out according to the actual situation, and congenital rubella syndrome (CRS) should be monitored. On the other hand, the current childhood immunization strategy should be maintained to ensure a high level of routine immunization coverage with two timely doses of RCV. School enrollment should also ensure that all children receive two doses of RCV. Furthermore, it is vital to strengthen the school's response capacity to rubella outbreaks. We believe this study has several substantial implications for future rubella prevention and control strategies in Anhui province.

## Data availability statement

The original contributions presented in the study are included in the article/Supplementary material, further inquiries can be directed to the corresponding authors.

## Ethics statement

Ethical review and approval was not required for the study on human participants in accordance with the local legislation and institutional requirements. Written informed consent from the participants' legal guardian/next of kin was not required to participate in this study in accordance with the national legislation and the institutional requirements.

## Author contributions

BS, ZL, and NZ were involved in the conception of the study. NZ, BW, XL, and JT analyzed the data and drafted the manuscript. XC, SZ, and YC were responsible for quality control of laboratory diagnosis of cases. All authors have contributed significantly to the final article and have approved it.

## Conflict of interest

The authors declare that the research was conducted in the absence of any commercial or financial relationships that could be construed as a potential conflict of interest.

## Publisher's note

All claims expressed in this article are solely those of the authors and do not necessarily represent those of their affiliated organizations, or those of the publisher, the editors and the reviewers. Any product that may be evaluated in this article, or claim that may be made by its manufacturer, is not guaranteed or endorsed by the publisher.
