# Supplementary material for: Epidemiological investigation and prevention and control strategies of rubella in Anhui province, China, from 2012 to 2021
Source: Front Public Health. 2022 Oct 6;10:991799. doi: 10.3389/fpubh.2022.991799 (PMC9583678; doi:10.3389/fpubh.2022.991799)
Supplement: Supplementary file 1 [file Data_Sheet_1.docx]

**TableS1** Number of rubella cases and non-cases by region in Anhui Province from 2012 to 2021

| Year |  | Cases |  |  |  | Non-cases |  |
| --- | --- | --- | --- | --- | --- | --- | --- |
|  | Northern Anhui | Central Anhui | Southern Anhui |  | Northern Anhui | Central Anhui | Southern Anhui |
| 2012 | 435 | 959 | 435 |  | 25521605 | 22338345 | 11818238 |
| 2013 | 142 | 537 | 177 |  | 25615284 | 22281377 | 11982475 |
| 2014 | 58 | 134 | 79 |  | 25793139 | 22437955 | 12066635 |
| 2015 | 307 | 255 | 273 |  | 26009606 | 22652350 | 12166214 |
| 2016 | 66 | 65 | 150 |  | 27393614 | 21113020 | 12929085 |
| 2017 | 4 | 22 | 14 |  | 27688896 | 21296982 | 12969586 |
| 2018 | 27 | 60 | 13 |  | 28010809 | 21484115 | 13052976 |
| 2019 | 156 | 449 | 105 |  | 28261214 | 21729893 | 13244159 |
| 2020 | 13 | 14 | 5 |  | 28380000 | 21931940 | 13347002 |
| 2021 | 6 | 20 | 7 |  | 26821793 | 21915901 | 12289462 |
| Total | 1214 | 2515 | 1258 |  | 269495960 | 219181878 | 125865832 |

**TableS2** Number of rubella cases and non-cases between male and female from 2012 to 2021

| Year | cases | |  | Non-cases | |
| --- | --- | --- | --- | --- | --- |
|  | Male | Female |  | Male | Female |
| 2012 | 1116 | 713 |  | 30094785 | 29583403 |
| 2013 | 541 | 315 |  | 30195210 | 29683926 |
| 2014 | 174 | 97 |  | 30555526 | 29742203 |
| 2015 | 516 | 319 |  | 30217779 | 30610391 |
| 2016 | 189 | 92 |  | 30907346 | 30528373 |
| 2017 | 18 | 22 |  | 31562463 | 30393001 |
| 2018 | 65 | 35 |  | 31586934 | 30960966 |
| 2019 | 458 | 252 |  | 31871523 | 31363743 |
| 2020 | 20 | 12 |  | 32154958 | 31503984 |
| 2021 | 21 | 12 |  | 31103369 | 29923787 |
| Total | 3118 | 1869 |  | 310249893 | 304293777 |

Table S3 Reported routine immunization coverage rate (%) of RCV1 in each city from 2009 to 2021

| **City** | |  |  |  |  |  | **Year** |  |  |  |  |  |  |  |
| --- | --- | --- | --- | --- | --- | --- | --- | --- | --- | --- | --- | --- | --- | --- |
|  | | 2009 | 2010 | 2011 | 2012 | 2013 | 2014 | 2015 | 2016 | 2017 | 2018 | 2019 | 2020 | 2021 |
| Hefei | 37.82 | | 79.42 | 92.08 | 94.92 | 96.89 | 97.75 | 97.64 | 98.53 | 98.68 | 98.87 | 99.04 | 99.13 | 99.1 |
| [Wuhu](http://192.168.140.15:8010/yb/queryInitSZ.do?query=change&action=general) | 37.71 | | 82.79 | 94.24 | 94.53 | 96.74 | 97.64 | 97.96 | 98.67 | 98.78 | 98.92 | 99.17 | 99.38 | 99.45 |
| [Bengbu](http://192.168.140.15:8010/yb/queryInitSZ.do?query=change&action=general) | 35.57 | | 71.51 | 83.7 | 89.98 | 94.06 | 96.13 | 97.21 | 98.42 | 98.66 | 99.02 | 99.27 | 99.49 | 99.37 |
| [Huainan](http://192.168.140.15:8010/yb/queryInitSZ.do?query=change&action=general) | 44.78 | | 80.29 | 90.34 | 93.74 | 96.19 | 97.49 | 98.07 | 98.73 | 98.99 | 99.19 | 99.41 | 99.61 | 99.52 |
| [Ma’anshan](http://192.168.140.15:8010/yb/queryInitSZ.do?query=change&action=general) | 42.91 | | 85.62 | 93.54 | 96.5 | 97.78 | 98.29 | 98.65 | 98.77 | 98.76 | 98.9 | 99.2 | 99.42 | 99.5 |
| [Huaibei](http://192.168.140.15:8010/yb/queryInitSZ.do?query=change&action=general) | 37.76 | | 78.81 | 87.68 | 91.86 | 95.16 | 96.28 | 96.08 | 97.59 | 98.16 | 98.65 | 99.13 | 99.42 | 99.66 |
| [Tongling](http://192.168.140.15:8010/yb/queryInitSZ.do?query=change&action=general) | 33.91 | | 86.88 | 94.45 | 96.15 | 97.66 | 98.25 | 98.59 | 99.08 | 99.02 | 99.24 | 99.44 | 99.68 | 99.7 |
| [Anqing](http://192.168.140.15:8010/yb/queryInitSZ.do?query=change&action=general) | 42.65 | | 82.03 | 91.23 | 93.89 | 96.19 | 97.49 | 97.82 | 98.83 | 98.92 | 99.1 | 99.32 | 99.52 | 99.53 |
| [Huangshan](http://192.168.140.15:8010/yb/queryInitSZ.do?query=change&action=general) | 62.36 | | 92.36 | 96.79 | 97.85 | 98.63 | 98.95 | 99.29 | 99.39 | 99.44 | 99.34 | 99.55 | 99.74 | 99.73 |
| [Chuzhou](http://192.168.140.15:8010/yb/queryInitSZ.do?query=change&action=general) | 34.47 | | 76.59 | 88.93 | 92.18 | 95.28 | 96.55 | 97.46 | 98.23 | 98.49 | 98.63 | 99.1 | 99.42 | 99.54 |
| [Fuyang](http://192.168.140.15:8010/yb/queryInitSZ.do?query=change&action=general) | 37.95 | | 71.32 | 83.81 | 89.36 | 93.66 | 95.9 | 97.46 | 98.4 | 98.83 | 99.05 | 99.28 | 99.48 | 99.47 |
| [Suzhou](http://192.168.140.15:8010/yb/queryInitSZ.do?query=change&action=general) | 48.06 | | 81.54 | 90.98 | 94.85 | 97 | 97.96 | 98.67 | 99.09 | 99.3 | 99.43 | 99.59 | 99.75 | 99.76 |
| [Lu’an](http://192.168.140.15:8010/yb/queryInitSZ.do?query=change&action=general) | 42.18 | | 78.93 | 89.78 | 93.09 | 95.64 | 97.05 | 97.89 | 98.52 | 98.62 | 98.91 | 99.05 | 99.33 | 99.35 |
| [Bozhou](http://192.168.140.15:8010/yb/queryInitSZ.do?query=change&action=general) | 33.3 | | 67.93 | 81.33 | 86.76 | 91.45 | 94.68 | 96.14 | 97.52 | 98.18 | 98.56 | 98.94 | 99.47 | 99.53 |
| [Chizhou](http://192.168.140.15:8010/yb/queryInitSZ.do?query=change&action=general) | 46.21 | | 84.55 | 92.2 | 94.35 | 96.42 | 97.35 | 98.21 | 98.66 | 98.93 | 99.16 | 99.29 | 99.61 | 99.66 |
| [Xuancheng](http://192.168.140.15:8010/yb/queryInitSZ.do?query=change&action=general) | 55.64 | | 88.45 | 94.42 | 96.1 | 97.59 | 98.4 | 98.65 | 98.88 | 99.17 | 99.11 | 99.38 | 99.64 | 99.72 |

Table S4. Reported routine immunization coverage rate (%) of RCV2 in each city from 2009 to 2021

| **City** |  |  |  |  |  | **Year** |  |  |  |  |  |  |  |
| --- | --- | --- | --- | --- | --- | --- | --- | --- | --- | --- | --- | --- | --- |
|  | 2009 | 2010 | 2011 | 2012 | 2013 | 2014 | 2015 | 2016 | 2017 | 2018 | 2019 | 2020 | 2021 |
| [Hefei](http://192.168.140.15:8010/yb/queryInitSZ.do?query=change&action=general) | 14.82 | 41.25 | 67.93 | 74.7 | 79.84 | 84.37 | 85.88 | 94.32 | 92.19 | 94.57 | 95.51 | 95.22 | 97.88 |
| [Wuhu](http://192.168.140.15:8010/yb/queryInitSZ.do?query=change&action=general) | 1.93 | 50.43 | 73.39 | 70.18 | 77.21 | 82.34 | 85.39 | 92.24 | 91.56 | 93.99 | 94.57 | 95.36 | 98.48 |
| [Bengbu](http://192.168.140.15:8010/yb/queryInitSZ.do?query=change&action=general) | 94.93 | 45.93 | 65.96 | 81.08 | 88.77 | 92.24 | 95 | 96.93 | 97.89 | 98.35 | 98.78 | 98.99 | 99.04 |
| [Huainan](http://192.168.140.15:8010/yb/queryInitSZ.do?query=change&action=general) | 93.33 | 58.32 | 79.38 | 88.39 | 92.93 | 94.82 | 96.9 | 97.88 | 98.49 | 98.73 | 99.04 | 99.21 | 99.36 |
| [Ma’anshan](http://192.168.140.15:8010/yb/queryInitSZ.do?query=change&action=general) | 37.24 | 55.69 | 81.06 | 88.01 | 91.96 | 93.76 | 95.37 | 97.21 | 97.37 | 97.64 | 98.09 | 98.19 | 99.12 |
| [Huaibei](http://192.168.140.15:8010/yb/queryInitSZ.do?query=change&action=general) | 8.27 | 41.67 | 66.51 | 65.63 | 75.26 | 79.75 | 83.82 | 92.28 | 94.89 | 94.69 | 96.1 | 95.86 | 98.62 |
| [Tongling](http://192.168.140.15:8010/yb/queryInitSZ.do?query=change&action=general) | 1.37 | 49.39 | 75.52 | 74.22 | 79.89 | 84.15 | 86.6 | 92.95 | 92.41 | 94.85 | 96.94 | 96.36 | 98.88 |
| [Anqing](http://192.168.140.15:8010/yb/queryInitSZ.do?query=change&action=general) | 7.66 | 50.19 | 68.43 | 75.7 | 81.83 | 86.19 | 89.23 | 93.9 | 93.38 | 94.92 | 95.95 | 96.67 | 98.61 |
| [Huangshan](http://192.168.140.15:8010/yb/queryInitSZ.do?query=change&action=general) | 50.11 | 69.88 | 85.77 | 90.28 | 92.4 | 93.84 | 94.95 | 97.08 | 97.28 | 97.25 | 97.68 | 97.88 | 99.23 |
| [Chuzhou](http://192.168.140.15:8010/yb/queryInitSZ.do?query=change&action=general) | 13.35 | 41.84 | 67.34 | 71.66 | 79.49 | 84.53 | 87.91 | 93.03 | 93.59 | 95.06 | 96.34 | 96.71 | 98.92 |
| [Fuyang](http://192.168.140.15:8010/yb/queryInitSZ.do?query=change&action=general) | 15.35 | 41.7 | 63.02 | 76.59 | 83.89 | 87.84 | 91.7 | 94.53 | 95.59 | 96.7 | 97.72 | 98.16 | 99.01 |
| [Suzhou](http://192.168.140.15:8010/yb/queryInitSZ.do?query=change&action=general) | 20.14 | 43.82 | 66.81 | 77.8 | 83.5 | 86.91 | 89.95 | 93.4 | 95.39 | 96.23 | 96.66 | 96.68 | 98.73 |
| [Lu’an](http://192.168.140.15:8010/yb/queryInitSZ.do?query=change&action=general) | 52.29 | 46.61 | 72.24 | 82.76 | 89.33 | 92.36 | 95.04 | 96.68 | 97.26 | 97.81 | 98.29 | 98.6 | 98.9 |
| [Bozhou](http://192.168.140.15:8010/yb/queryInitSZ.do?query=change&action=general) | 30.81 | 36.88 | 61.8 | 69.77 | 77.93 | 83.56 | 87.7 | 92.38 | 93.42 | 95.2 | 96.51 | 97.17 | 98.63 |
| [Chizhou](http://192.168.140.15:8010/yb/queryInitSZ.do?query=change&action=general) | 96.04 | 57.23 | 82.54 | 90.23 | 93.75 | 94.94 | 97.3 | 98.05 | 98.52 | 98.56 | 98.77 | 98.91 | 99.29 |
| [Xuancheng](http://192.168.140.15:8010/yb/queryInitSZ.do?query=change&action=general) | 33.02 | 58.03 | 77.73 | 80.62 | 85.35 | 88.71 | 89.63 | 93.68 | 94.44 | 95.06 | 95.85 | 96.12 | 98.65 |
